# ﻿*Temochloa* (Poaceae, Bambusoideae), a newly-recorded bamboo genus for China and Vietnam, with new taxa and a re-interpretation of flowering structures

**DOI:** 10.3897/phytokeys.246.129035

**Published:** 2024-09-04

**Authors:** Zhuo-Yu Cai, You-Yuan Zhang, Yi-Hua Tong, Tien Chinh Vu, Nian-He Xia

**Affiliations:** 1 Guangdong Provincial Key Laboratory of Digital Botanical Garden, South China Botanical Garden, Chinese Academy of Sciences, Guangzhou 510650, China Chinese Academy of Sciences Guangzhou China; 2 Key Laboratory of National Forestry and Grassland Administration on Plant Conservation and Utilization in Southern China, Guangzhou 510650, China Key Laboratory of National Forestry and Grassland Administration on Plant Conservation and Utilization in Southern China Guangzhou China; 3 South China National Botanical Garden, Guangzhou 510650, China South China National Botanical Garden Guangzhou China; 4 University of Chinese Academy of Sciences, Beijing 100049, China University of Chinese Academy of Sciences Beijing China; 5 Guiyang Vocational and Technical College, Guiyang 550081, China Guiyang Vocational and Technical College Guiyang China; 6 Vietnam National Museum of Nature, Vietnam Academy of Science and Technology, Hanoi 100803, Vietnam Vietnam National Museum of Nature, Vietnam Academy of Science and Technology Hanoi Vietnam; 7 Graduate University of Science and Technology, Vietnam Academy of Science and Technology, Hanoi 100803, Vietnam Graduate University of Science and Technology Hanoi Vietnam

**Keywords:** Bambusoideae, morphology, pseudospikelet, taxonomy

## Abstract

*Neomicrocalamus* and *Temochloa* are closely-related genera for which ‘inflorescence’ structures were incompletely understood and difficult to reconcile. After re-examining the inflorescence morphology, the so-called ‘spikelets’ of both genera as described should instead be recognised as pseudospikelets with mostly inactive axillary buds. The new bamboo taxa, comprising two varieties of a new species, are placed in *Temochloa*, representing a new genus record for China and Vietnam.

## ﻿Introduction

*Neomicrocalamus* Keng f. and *Temochloa* S. Dransf. are two climbing bamboo genera (Poaceae, Bambusoideae, Bambuseae) only distributed in limestone areas (Dransfield 2000; Li and Stapleton 2006; BPG 2012). Phylogenetically, they are sister groups (Ruiz-Sanchez and Sosa 2015; Zhou et al. 2017). In morphology, *Neomicrocalamus* possesses some characters in common with *Temochloa*, such as short-necked pachymorph rhizomes, scrambling culms and even the ambiguous inflorescence structures (Dransfield 2000; Li and Stapleton 2006).

When Keng (1983) established the genus *Neomicrocalamus*, he considered that the ‘inflorescence’ is semelauctant (determinate, characterised by true spikelets), meaning it completes its development within a single grand period of growth (McClure 1966). However, at the same time, he considered that the inflorescence basic unit is a pseudospikelet (typically with basal bracts subtending buds that will produce similarly branching lateral units, these then repeating the process, giving what has been called an iterauctant or indeterminate development; McClure (1934, 1966)), as indeed the basal prophyll and bracts are present. Stapleton (1994) held the same opinion as Keng. These descriptions are very confusing, as the semelauctant and iterauctant conditions would seem to be fundamentally contrasting. In the protologue of *Temochloa*, it was diagnosed as having a determinate ‘inflorescence with bracteate and prophyllate branches’ (Dransfield 2000).

With some newly-collected flowering material from China and Vietnam, it becomes possible for us to re-examine the ‘inflorescence’ structure of *Neomicrocalamus* and *Temochloa*. Furthermore, during the examination, we recognised that some taxa are new to science. These taxa are described and illustrated here.

## ﻿Materials and methods

Flowering material was dissected under a stereomicroscope (Mshot-MZ101) and images were taken with the camera attachment (Mshot-MSX2). Morphological comparisons and descriptions were based on the relevant literature including protologues, as well as herbarium specimens and living plants.

## ﻿Results

The newly-discovered bamboo plants are characterised by short-necked pachymorph rhizomes, scrambling culms, solitary and almost circular primary branch buds, branch complement with many short and subequal branches with an occasional dominant central branch that reiterates and approaches the size of the culm, pseudospikelets with 2–4 fertile florets; 6 stamens with emarginate anther apices, 3 stigmas and caryopses.

## ﻿Discussion

### ﻿Flowering structures in the three groups

The pseudospikelet should be interpreted as a condensed flowering branch which terminates in a spikelet proper and is basally supplied with a prophyll and bracts (reduced sheaths) subtending buds (McClure 1934, 1966). Typically, these subtended buds are able to develop as secondary pseudospikelets, which in turn lead to higher-order lateral pseudospikelets. This is termed an iterauctant or indeterminate development (McClure 1966). In contrast, a semelauctant or determinate development refers to a single episode of development of the flowering branch, conventionally associated with true spikelets (McClure 1966). However, it should be noted that McClure (1966) mentioned that the axillary buds of primary pseudospikelets of *Arundinariaprainii* Gamble, equivalent to *Neomicrocalamusprainii* (Gamble) Keng f., may remain dormant. Perhaps because of this, Keng (1983) employed the term ‘semelauctant’ for the ‘inflorescence’ of *Neomicrocalamus*. Afterwards, in the *Flora Reipublicae Popularis Sinicae*, inflorescence development was not discussed, but it was stated that, although axillary buds of the pseudospikelet type are present, they have never been known to develop as secondary pseudospikelets (Geng and Wang 1996). Probably for much the same reason, the ‘inflorescence’ of *Temochloa* was also regarded as being of a determinate nature, but its description in the protologue states it is “usually comprising one spikelet, in inflorescences with more than one spikelet . . . the axis [is]. . . terminated by a spikelet and a single branch . . . borne at each node, subtended by a bract/sheath and a prophyll . . .” (Dransfield 2000). It should be noted that Dransfield (2000) avoided using the term ‘semelauctant’ in her very careful description.

**Figure 1.**
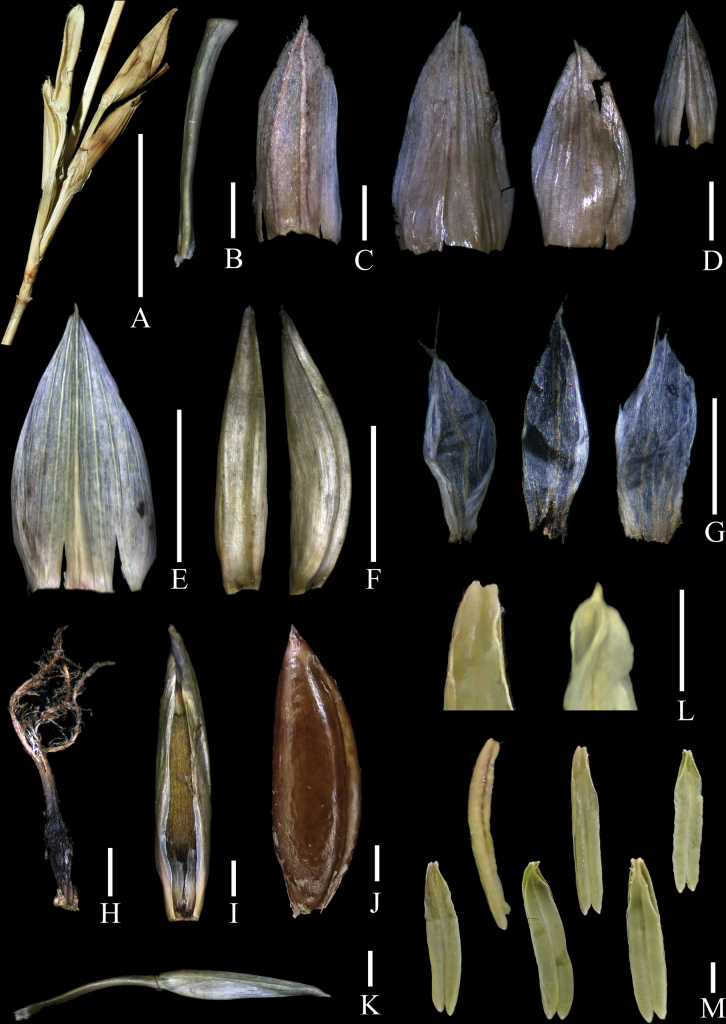
Floral morphology of *Temochloaelegans***A** pseudospikelets of *Neomicrocalamus* sp. showing the secondary pseudospikelet (left) developed from the base of the primary pseudospikelet (right) **B** rachilla segment **C** prophyll (abaxial view) **D** bracts (abaxial view) **E** lemma (abaxial view) **F** palea (left: back view; right: side view) **G** lodicules **H** pistil **I** young fruit subtended by a palea **J** mature fruit **K** apical not fully developed floret **L** anther apices (left from *Temochloaelegans*; right from *Neomicrocalamus* sp. included for comparison) **M** anthers.

In fact, according to our observation, pseudospikelets of *Neomicrocalamus* do produce secondary pseudospikelets, although it happens only occasionally (Fig. 1A). Therefore, the pseudospikelet development of *Neomicrocalamus* is still iterauctant rather than semelauctant, the latter conventionally linked to true spikelets. It would be confusing if the semelauctant condition is also said to develop pseudospikelets. Hence, the ‘spikelets’ of both *Neomicrocalamus* and *Temochloa* could be properly recognised as a variant (possibly extreme reduction) of the typical pseudospikelet structure, in which axillary buds are not as active as in the typical condition.

### ﻿The new species of *Temochloa*

The morphological characters of our newly-discovered bamboos are more similar to *Temochloa*. The primary branch buds of both the newly-discovered bamboos and *Temochloa* are nearly circular and their culm leaf sheaths are shallowly grooved, whereas *Neomicrocalamus* has lanceolate buds and plane culm leaf sheaths (Dransfield 2000; Li and Stapleton 2006). Besides, the anther apices of the newly-discovered bamboos are emarginate (Fig. 1L, left), while those of *Neomicrocalamus* are conspicuously cuspidate due to prolongation of the connective (Fig. 1L, right). However, to date, some characters, including stamens, of *Temochloa* are still unknown.

On the other hand, the newly-discovered bamboos and *Temochloa* occur at very low elevations, 50–250 m, rarely reaching 700 m, whereas *Neomicrocalamus* taxa have hitherto only been found above 1000 m (Dransfield 2000; Li and Stapleton 2006).

The similar morphology and distribution, in terms of elevation, of the newly-discovered bamboos and *Temochloa* are also commensurate with their close phylogenetic affinities. The phylogenetic evidence indicates that the newly-discovered bamboos originate from introgressive hybridisation between *Temochloaliliana* S. Dransf. and *N.prainii* (Cai et al. 2023; the newly-discovered bamboo accessions referred as *BH85*, *2018VNB018* and *2018VNB040*). The newly-discovered bamboos inherited most (80.7%) of its genome from *T.liliana*; therefore, genetically, these two groups are closer related (Cai et al. 2023).

Given the present evidence accrued from a combination of morphological, phylogenetic and biogeographical evidence, we propose that the newly-discovered bamboos are an undescribed species with a variety best placed in *Temochloa*, which then represents a newly-recorded genus for both China and Vietnam.

### ﻿Taxonomic treatment

#### ﻿ Temochloa elegans

Taxon classificationPlantaePoalesPoaceae

N.H.Xia, Y.Y.Zhang, Z.Y.Cai & Y.H.Tong sp. nov.

2112448B-9986-5C10-A030-9A32BEA71BD3

urn:lsid:ipni.org:names:77347888-1

Figs 1 , 2

##### Diagnosis.

The new species resembles *Temochloaliliana*, but differs by its subsolid (vs. hollow) culm internodes, hairy (vs. glabrous) prophylls of the pseudospikelets, paleae longer than (vs. as long as) lemmas and acute to slightly obtuse (vs. 2-lobed) palea apices.

##### Type.

China, Guangxi, Jingxi, near Bandan Village, limestone, 22°53'23"N, 106°21'16"E, 703 m elev., fl. (floret, flower), fr. (fructus, fruit), 10 June 2020, *N.H. Xia et al. BH85* (holotype: IBSC!).

**Figure 2.**
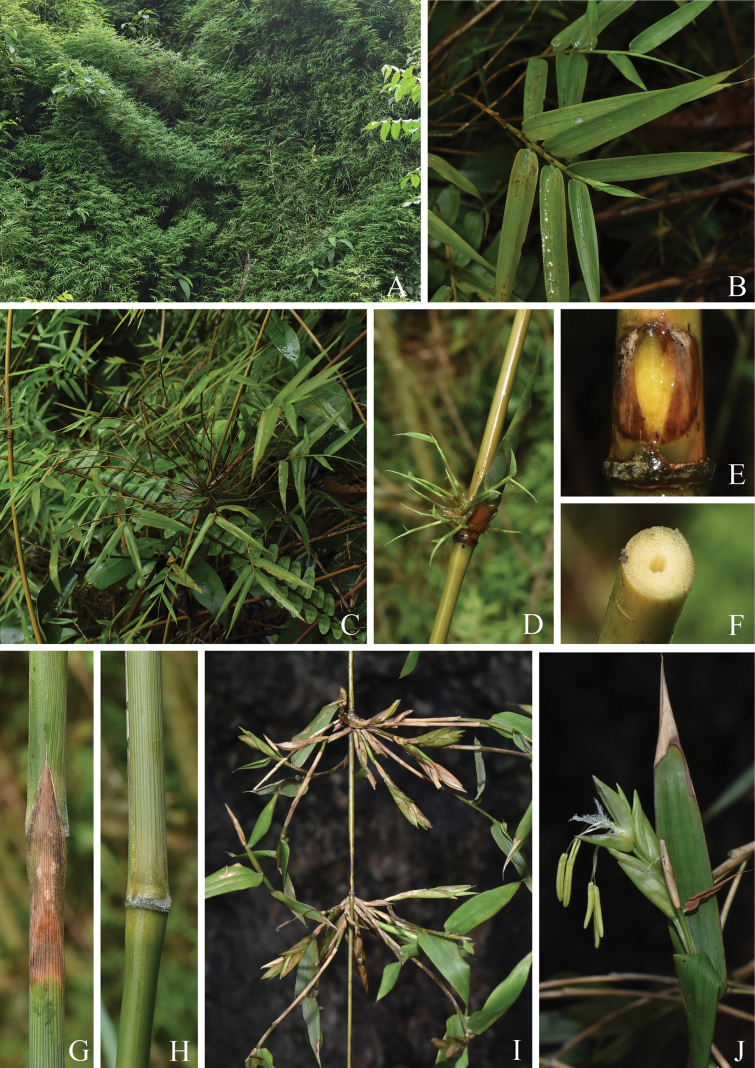
Morphology of *Temochloaelegans***A** habit **B** foliage leaves **C** branch complement without a dominant central branch **D** branch complement with a developing dominant central branch **E** primary branch bud **F** culm internode section **G** culm leaf (upper half) **H** culm leaf (lower half) **I** flowering branches **J** spikelet proper.

##### Description.

Clumps unicaespitose, open, spreading. Rhizomes short-necked, pachymorph. Culms scrambling; internodes subsolid, 15–30(–35) cm long, 4–5 mm diam., glabrous; supranodal ridges slightly prominent; sheath scar prominent with a persistent sheath-base collar. Primary branch bud solitary, nearly circular, compressed, 7–8 mm long, puberulent, the lateral edges ciliolate. Culm leaf sheath deciduous, narrowly triangular, green when young, margins glabrous, abaxial surface glabrous and white pubescent at the base, with shallow longitudinal grooves, grooves with white pubescence becoming glabrous; ligule inconspicuous; auricles and oral setae absent; culm leaf blade persistent, erect, acicular. Branches intravaginal, many and subequal at each node, central branch sometimes dominant, reiterating and approaching the size of the culm. Foliage leaves 6–13 per ultimate branch; foliage leaf sheath pubescent, margins glabrous; ligule truncate, no longer than 0.5 mm high, puberulent, ciliolate; auricles and oral setae absent; foliage leaf blades papery, lanceolate to oblong, 5–8 cm long, 0.4–0.8 cm wide, abaxial surface glabrous, adaxial surface (sub)glabrous, one margin entire, the other serrulate, base rounded-obtuse to rounded-truncate, apex acicular, acuminate, secondary longitudinal veins 2–3 pairs, transverse veins inconspicuous.

Pseudospikelets solitary, secondary pseudospikelets rarely produced, slightly compressed, prophyll oblong, 3–3.5 mm long, papery, 2-keeled, keels ciliate, puberulent between keels, adaxial surface apically puberulent, apex acute; bracts 4–5, papery to leathery, gemmiferous or not, triangular to lanceolate, 2.5–5 mm long, abaxial surface glabrous or puberulent above the middle, adaxial surfaces glabrous or apically puberulent, 7–9-veined, apex acute and mucronate; fertile florets 2–4, uppermost floret not fully developed; rachilla segments compressed, 4–5 mm long, glabrous; glumes absent; lemma leathery, lanceolate, ca. 6–8 mm long, both surfaces glabrous or adaxial surface apically puberulent, 9–11-veined, apex acute and mucronate, callus inconspicuous, no more than 0.5 mm long, glabrous; palea longer than lemma, ca. 7–9 mm long, glabrous, 2-keeled, 2–3-veined between keels, each side 3–4-veined, apex acute to slightly obtuse; lodicules 3, the anterior two, broadly ovate, ca. 2 mm long, 3–4-veined, the posterior one, lanceolate, ca. 2 mm long, 1–3-veined; stamens 6, filaments free, anthers yellow, ca. 3.5–4.5 mm long, apex emarginate; stigmas 3, 2–2.5 mm long, plumose, ovary ellipsoid, ca. 1.5 mm long. Caryopsis ellipsoid, ca. 7–8 mm long.

##### Phenology.

New shoots around May.

##### Etymology.

The specific epithet refers to its elegant habit.

##### Chinese name.

雅竹 (yǎ zhú).

##### Distribution and habitat.

This species occurs in the limestone area of southwest Guangxi, China and northeast Vietnam, at elevations of 210(–700) m.

##### Conservation status.

Up to now, *T.elegans* is known from only two locations in China and Vietnam. It is not very common at those locations so the number of mature clumps appears to be limited. The Vietnamese population is well protected in the Nature Reserve, while the Chinese population is distributed along the highway and not in any protected area. It should probably be categorised as Near Threatened (NT) (IUCN Standards and Petitions Committee 2022).

##### Additional specimens examined

**(paratypes).** Vietnam, Bac Kan, Ba Be Lake, limestone, 22°25'15"N, 105°36'53"E, 170 m elev., 20 May 2018, *N.H. Xia* et al. *2018VNB018* (IBSC!, VNMN!).

#### ﻿ Temochloa elegans var. glabra

Taxon classificationPlantaePoalesPoaceae

N.H.Xia, Z.Y.Cai, Y.Y.Zhang & J.B.Ni var. nov.

2C91FF53-FEBE-5F33-9A83-B2030E4EE784

urn:lsid:ipni.org:names:77347889-1

##### Diagnosis.

This variety can be differentiated from Temochloaelegansvar.elegans in its glabrous foliage leaf sheaths and glabrous foliage leaf ligules.

##### Type.

Vietnam, Ha Giang, Minh Ngoc Village, limestone, 22°43'19"N, 105°11'36"E, 210 m elev., 24 May 2018; *N.H. Xia et al. 2018VNB040* (holotype: IBSC!, isotype: VNMN!).

##### Etymology.

The specific epithet refers to its glabrous foliage leaf sheaths and glabrous foliage leaf ligules.

##### Chinese name.

光雅竹 (guāng yǎ zhú).

##### Distribution and habitat.

This species occurs in the limestone area of northeast Vietnam, at elevations of 140–210 m.

##### Conservation status.

Up to now, T.elegansvar.glabra is known from only one location in Vietnam. Less than 10 clumps were found. Due to the insufficient field survey, it should probably be categorised as Data Deficient (DD) (IUCN Standards and Petitions Committee 2022).

##### Additional specimens examined

**(paratypes).** Vietnam, Ha Giang, Minh Ngoc Village, limestone, 22°43'48"N, 105°12'25"E, 140 m elev., 2018 *N.H. Xia* et al. *2018VNB032* (IBSC!, VNMN!)

## Supplementary Material

XML Treatment for Temochloa elegans

XML Treatment for Temochloa elegans var. glabra

## ﻿Additional information

### Author ORCIDs

Zhuo-Yu Cai https://orcid.org/0000-0001-9288-0882

You-Yuan Zhang https://orcid.org/0000-0002-8463-147X

Yi-Hua Tong https://orcid.org/0000-0002-5034-005X

Tien Chinh Vu https://orcid.org/0000-0002-5014-8500

Nian-He Xia https://orcid.org/0000-0001-9852-7393

### Data availability

All of the data that support the findings of this study are available in the main text.
